# Verordnungshäufigkeit von physikalischer Therapie bei entzündlich rheumatischen Erkrankungen

**DOI:** 10.1007/s00393-022-01180-x

**Published:** 2022-03-22

**Authors:** Katinka Albrecht, Ursula Marschall, Angela Zink, Johanna Callhoff

**Affiliations:** 1 Programmbereich Epidemiologie und Versorgungsforschung, Deutsches Rheuma-Forschungszentrum Berlin, Charitéplatz 1, 10117 Berlin, Deutschland; 2 Abteilung Medizin und Versorgungsforschung, Barmer, Wuppertal, Deutschland; 3 Institut für Sozialmedizin, Epidemiologie und Gesundheitsökonomie, Charité Universitätsmedizin Berlin, Berlin, Deutschland

**Keywords:** Versorgung, Physiotherapie, Abrechnungsdaten, Arthritis, Beweglichkeit, Health care, Physiotherapy, Claims data, Arthritis, Mobility

## Abstract

**Zielsetzung:**

Es erfolgte die Untersuchung der Verordnungshäufigkeit von ambulanter physikalischer Therapie (PT) bei Versicherten mit der Diagnose einer rheumatoiden Arthritis (RA), axialen Spondyloarthritis (axSpA), Psoriasisarthritis (PsA) oder eines systemischen Lupus erythematodes (SLE) in den Jahren 2005 bis 2020.

**Methodik:**

Eingeschlossen wurden erwachsene Versicherte der BARMER Krankenversicherung mit Diagnose einer RA (ICD-10: M05, M06), axSpA (M45), PsA (M07.0-3) oder SLE (M32.1,8,9). PT wurden über das bundeseinheitliche Positionsnummernverzeichnis für Heilmittelleistungen identifiziert. Berichtet wird der Anteil an Personen mit mindestens einer Verordnung von PT in den Jahren 2005 bis 2020 nach Alters- und Geschlechtergruppen. Außerdem wurden die Anzahl und die Dauer der Verschreibungen verglichen, und es wurde analysiert, ob Versicherte mit fachärztlichem Kontakt häufiger PT erhalten.

**Ergebnisse:**

Im Jahr 2020 erhielten 43 % (SLE), 46 % (RA, PsA) und 49 % (axSpA) der Versicherten mindestens eine PT-Verordnung. Am häufigsten wurde Krankengymnastik rezeptiert (37 %), gefolgt von manueller Therapie (14 %) und Thermotherapie (10 %). Seit 2005 hat sich der Anteil der Versicherten, die Krankengymnastik erhalten, nicht verändert. Manuelle Therapie wird zunehmend verordnet (+7 %), während Massagen (−10 %), Thermotherapie (−7 %) und Elektrotherapie (3 % in 2005, 2 % in 2020) rückläufig sind (Zahlen zu RA). Auch die Anzahl der Verschreibungen hat sich seit 2010 nicht wesentlich verändert. Versicherte in orthopädischer Betreuung erhalten häufiger PT als Versicherte in hausärztlicher oder internistisch-rheumatologischer Betreuung; 61- bis 80-jährige Patientinnen werden am häufigsten mit PT behandelt.

**Schlussfolgerung:**

Etwas weniger als die Hälfte aller Versicherten mit RA-, axSpA-, PsA- oder SLE-Diagnose erhalten eine ambulante Verordnung von PT. Dieser Anteil hat sich in den letzten 15 Jahren kaum verändert.

**Zusatzmaterial online:**

Die Online-Version dieses Beitrags (10.1007/s00393-022-01180-x) enthält die Tabellen S1–S3 und die Abbildungen S1–S2.

## Abstract (en)

**Objective:**

To investigate the frequency of outpatient physical therapy (PT) in persons with rheumatoid arthritis (RA), axial spondylarthritis (axSpA), psoriatic arthritis (PsA) or systemic lupus erythematosus (SLE) between 2005 and 2020.

**Methods:**

Adult insured persons of the BARMER health insurance fund with a diagnosis of RA (ICD-10: M05, M06), axSpA (M45), PsA (M07.0-3) or SLE (M32.1,8,9) were included. The prescription of PT was identified via the national item number index for therapeutic products. The proportion of persons with at least 1 prescription in the years 2005 to 2020 is reported as well as PT prescriptions by age and gender groups. In addition, the number and duration of prescriptions were compared and it was analyzed whether persons in specialist care received PT more frequently.

**Results:**

In 2020, 43% (SLE), 46% (RA, PsA) and 49% (axSpA) received at least 1 PT prescription. Physiotherapy was prescribed most frequently (37%), followed by manual therapy (14%) and thermotherapy (10%). Since 2005 the number of insured persons receiving PT has not changed. Manual therapy is increasingly prescribed (+7%), while massage (−10%), thermotherapy (−7%) and electrotherapy (3% in 2005, 2% in 2020) have been decreasing (data relating to RA). The number of prescriptions has not relevantly changed since 2010. Persons in orthopedic care received PT more frequently than persons in general or rheumatological care. Female patients 61–80 years old were most frequently treated with PT.

**Conclusion:**

Slightly less than half of all persons with an RA, axSpA, PsA or SLE diagnosis received outpatient PT. This proportion has hardly changed in the last 15 years.

Physikalische Therapie (PT), zu der die aktive Physiotherapie sowie passive Anwendungen gehören, ist ein zentraler Bestandteil der nichtmedikamentösen therapeutischen Versorgung von Patienten mit entzündlich rheumatischen Erkrankungen [1]. Bei Patienten mit rheumatoider Arthritis (RA), Psoriasisarthritis (PsA) und axialer Spondyloarthritis (axSpA) können PT-Behandlungen sowohl zu einer Schmerzlinderung und einer Lösung von Verspannungen beitragen, als auch die Beweglichkeit verbessern, die Muskulatur kräftigen und die Funktionalität stabilisieren. Auch Patientinnen mit einem systemischen Lupus erythematodes (SLE) profitieren von gezielten körperlichen Trainingsprogrammen, die das Allgemeinbefinden und die Beweglichkeit steigern und auch positive Effekte auf eine begleitende Depressivität haben [1].

Bei bestimmten Erkrankungen, zu denen auch die axSpA und der SLE zählen, ist eine langfristige Verordnung von Heilmitteln nach der Heilmittel-Richtlinie möglich. Über die Diagnoseliste des besonderen Versorgungsbedarfs ist auch die langfristige Verordnung von Heilmitteln bei Patienten mit RA und PsA festgelegt [2]. Die Kosten für diese Verordnungen werden seit 2012 extrabudgetär erfasst, sodass aus rechtlicher Sicht die Verordnung von PT für diese Krankheitsbilder über einen Zeitraum von mindestens 1 Jahr abgesichert ist. Es gab in den letzten Jahren Änderungen der Heilmittelverordnung [3], die dazu beitragen sollen, die Verordnung von PT bei chronisch entzündlichen Erkrankungen zu erleichtern. Die Fragestellung dieser Arbeit war, ob Verordnungen von PT bei entzündlich rheumatischen Erkrankungen in den letzten 15 Jahren zugenommen haben.

## Methodik

Für die Auswertung wurden ambulant-ärztliche Abrechnungsdaten der BARMER verwendet, die als gesetzliche Krankenversicherung bundesweit rund 9 Mio. Versicherte umfasst. Es wurden erwachsene Versicherte in den Jahren 2005 bis 2020 mit ICD-10-Diagnosen einer der folgenden entzündlich rheumatischen Erkrankungen – RA (M05, M06), axSpA (M45), PsA (M07.0-3) oder SLE (M32.1,8,9) – eingeschlossen. Dabei musste die Diagnose jeweils in mindestens 2 unterschiedlichen Quartalen für das jeweilige Jahr vorliegen.

Die Verordnung von PT und Ergotherapie wurde über das bundeseinheitliche Positionsnummern-Verzeichnis für Heilmittelleistungen (Massagen: X01, Krankengymnastik: X03–X10, manuelle Therapie: X12, Elektrotherapie: X13, Thermotherapie: X15, Ergotherapie X40–X45) identifiziert.

Verordnungen von nichtsteroidalen Antirheumatika (NSAR), Glukokortikoiden (GC), konventionell synthetischen „disease-modifying antirheumatic drugs“ (csDMARDs) und biologischen (b)DMARDs wurden über das anatomisch-therapeutisch-chemische Klassifikationssystem (ATC-Codes) identifiziert. Die entsprechenden ATC-Codes sind online in Tabelle S1 aufgeführt.

Komorbiditäten, die häufig mit einer Heilmittelverordnung einhergehen, werden nach ICD-10-Kodierung berichtet: Arthrosen (M15–M17), Osteoporose (M80–82), Fibromyalgie (M79.70) und Rückenschmerz (M50–54).

Ein Facharztkontakt wurde über die Angabe der Facharztgruppe in der lebenslangen Arztnummer identifiziert (Orthopäden 6, 10–12, internistische Rheumatologen 31, Allgemeinmediziner 1–3). Zusätzlich wurden Ärzte, die rheumatologische Abrechnungspositionen (13690, 13691,13692, 13700, 13701, 99012) verwendet haben, als internistische Rheumatologen berücksichtigt. Alle anderen Facharztgruppen wurden als „Sonstige“ zusammengefasst.

Ausgewertet wurde der Anteil an Personen mit mindestens einer Verschreibung der jeweiligen PT in den Jahren 2005 bis 2020, aufgeteilt nach Krankheitsbildern. Zusätzlich wird die Häufigkeit der Verordnungen nach Altersgruppen (18 bis 40, 41 bis 60, 61 bis 80, > 80 Jahre) und nach Geschlecht berichtet. In einer Sensitivitätsanalyse wurden die Ergebnisse auf 2 Standardpopulationen bezüglich Alters- und Geschlechtsstruktur standardisiert: erstens auf die deutsche Population der gesetzlich Krankenversicherten von 2020 [4] und zweitens auf die Versichertenpopulationen mit der jeweiligen Diagnose in 2020 (d. h. für RA wurden die Daten der Jahre 2005 bis 2019 auf die Alters- und Geschlechtsstruktur der Versicherten mit RA-Diagnose von 2020 standardisiert, analog für axSpA, PsA, SLE). Das Alter wurde dabei in 5‑Jahres-Schritten berücksichtigt (20 bis unter 25 Jahre, 25 bis unter 30 Jahre, …, 90 Jahre und älter).

Außerdem wurde die Anzahl an verordneten Einheiten in den Jahren 2010 und 2020 verglichen. Dazu wurde für alle Personen, die eine Verordnung mit einer PT erhalten haben, berechnet, wie viele Einheiten im Mittel im jeweiligen Jahr verordnet wurden.

## Ergebnisse

Aus dem Jahr 2020 wurden Daten von *n* = 138.984 (RA), *n* = 29.249 (axSpA), *n* = 19.760 (PsA) und *n* = 5458 (SLE) Versicherten der BARMER eingeschlossen. Das mittlere Alter lag zwischen 59 (SLE) und 69 Jahren (RA) und der Anteil weiblicher Personen zwischen 50 % (axSpA) und 89 % (SLE). Seit 2005 sind die Versichertenzahlen bei allen Krankheitsbildern kontinuierlich angestiegen (Tabelle S2 im Online-Zusatzmaterial). Das mittlere Alter hat sich in diesem Zeitraum um 4 Jahre erhöht. An Begleitdiagnosen, die häufig mit einer PT-Verordnung einhergehen, sind insbesondere Rückenschmerzen mit 39 % (SLE) bis 48 % (RA) und Arthrosen mit 24 % (SLE) bis 43 % (RA) häufig.

Glukokortikoide wurden häufig bei RA (40 %) und SLE (47 %) verordnet, ein csDMARD (inklusive Antimalariamittel) erhielten 36 % (RA) bis 59 % (SLE). Ein bDMARD wurde bei 6 % (SLE), 11 % (RA), 16 % (axSpA) und 26 % (PsA) verordnet und ein tsDMARD bei 3 % (RA) und 4 % (PsA). Alle Daten aus den Jahren 2005 und 2020 sind in Tab. 1 dargestellt.

**Table 1:** 

	RA		axSpA		PsA		SLE	
	2005	2020	2005	2020	2005	2020	2005	2020
*N*	78.834	144.921	15.448	29.249	2998	19.760	2569	5458
Weiblich (%)	81	78	45	50	65	67	91	89
Alter, MW (SD)	65 (14)	69 (14)	57 (14)	61 (15)	57 (13)	62 (13)	53 (15)	59 (15)
18 bis 40 Jahre	5	4	13	11	9	7	23	13
41 bis 60 Jahre	29	22	43	35	49	35	44	40
61 bis 80 Jahre	52	51	40	42	39	50	30	38
81+ Jahre	13	23	5	12	3	8	3	9
*Begleitdiagnosen (%)*								
Arthrose	30	43	19	29	27	37	14	24
Osteoporose	21	24	11	13	12	15	20	23
Fibromyalgie	0,7	1,7	0,6	5,1	1,0	7,2	0,8	7,4
Rückenschmerz	41	48	37	47	39	47	30	39
*Medikamentöse Therapie (%)*								
Glukokortikoide	37	40	15	18	34	30	60	47
NSAR	59	43	60	49	64	48	42	32
csDMARDs	33	36	11	11	48	40	54	59
bDMARDs	3	11	3	16	6	26	1	6
tsDMARDs	–	3	–	0,4	–	4	–	1

*RA* rheumatoide Arthritis, *asSpA* axiale Spondyloarthritis, *PsA* Psoriasisarthritis, *SLE* systemischer Lupus erythematodes, *MW* Mittelwert, *SD* Standardabweichung, *NSAR* nichtsteroidale Antirheumatika, *csDMARDs* konventionell synthetische „disease-modifying antirheumatic drugs“, *bDMARDs* biologische „disease-modifying antirheumatic drugs“, *tsDMARDs* „targeted synthetic disease-modifying antirheumatic drugs“

### Verordnungen von physikalischer Therapie

Es hatten 43 % (SLE) bis 49 % (axSpA) der Versicherten mindestens 1 PT-Verordnung im Jahr 2020. Am häufigsten wurde Krankengymnastik verordnet, gefolgt von manueller Therapie und Wärme/-Kältetherapie. Thermotherapie wurde am häufigsten bei SLE verordnet (19 %); 8–11 % der Versicherten erhielten eine Ergotherapie. Aufgrund der Pandemie in 2020 sind auch die Werte von 2019 angeführt, diese sind geringfügig höher als in 2020 (Tab. 2).

**Table 2:** 

	RA		axSpA		PsA		SLE	
	2019	2020	2019	2020	2019	2020	2019	2020
Krankengymnastik	39	37	40	38	38	36	34	34
Massagen	3	3	3	3	3	2	3	3
Manuelle Therapie	15	14	17	16	17	16	15	14
Thermotherapie	11	10	12	11	13	11	10	19
Elektrotherapie	2	2	2	2	2	2	2	2
Mindestens 1 PT	49	46	51	49	49	46	45	43
Ergotherapie	11	11	8	8	10	11	11	9

*RA* rheumatoide Arthritis, *axSpA* axiale Spondyloarthritis, *PsA* Psoriasisarthritis, *SLE* systemischer Lupus erythematodes, *PT* physikalische Therapie

### Verordnungstrends von physikalischer Therapie 2005 bis 2020

**Figure 1:**
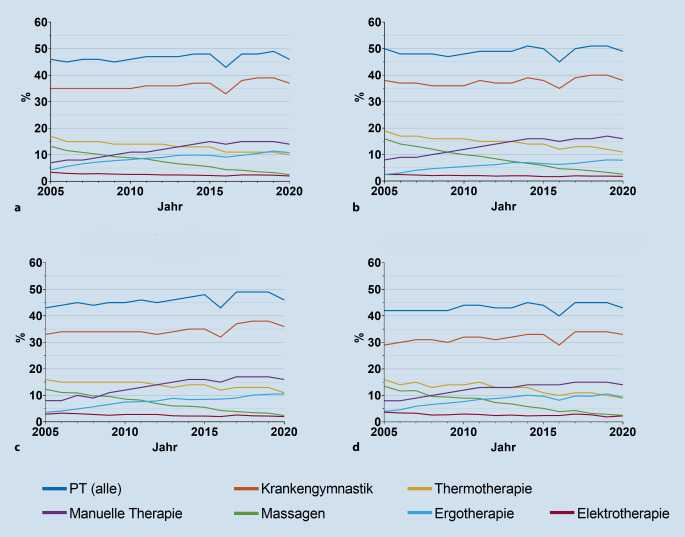

Seit 2015 ist der Anteil an Versicherten, die Krankengymnastik erhalten, konstant. Die Verordnung von Massagen hat kontinuierlich abgenommen, während manuelle Therapie und Ergotherapie häufiger verordnet werden (Abb. 1). Die einzelnen Werte sind für alle Krankheitsbilder in der Tabelle S2 berichtet. Standardisiert man diese Daten auf die GKV(gesetzliche Krankenversicherung)-Gesamtpopulation von 2020 oder auf die Barmer-Versichertenpopulation mit der jeweiligen Diagnose von 2020, ergibt sich kaum ein Unterschied (Abbildungen S1 und S2).

### Verordnungen von physikalischer Therapie nach Alter und Geschlecht

Frauen erhielten häufiger PT-Verordnungen als Männer, dies trifft für alle Krankheitsbilder und für alle Altersgruppen zu. Versicherte über 60 Jahre hatten häufiger PT-Verordnungen als jüngere Versicherte (Tab. 3).

**Table 3:** 

	RA	axSpA	PsA	SLE
Männer	37	43	36	34
Frauen	49	55	52	44
18 bis 40 Jahre	34	41	32	30
41 bis 60 Jahre	43	49	45	40
61 bis 80 Jahre	47	49	48	47
≥ 81 Jahre	50	54	54	53

*RA* rheumatoide Arthritis, *axSpA* axiale Spondyloarthritis, *PsA* Psoriasisarthritis, *SLE* systemischer Lupus erythematodes, *PT* physikalische Therapie

### Trends der Verordnung von physikalischen Therapien nach Alter und Geschlecht

**Figure 2:**
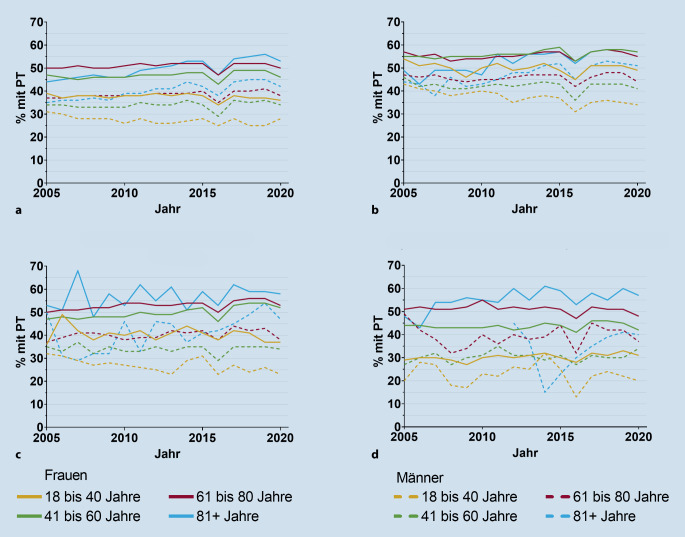

In der Altersgruppe der 18- bis 40-Jährigen sind die Verordnungen rückläufig, während sie bei den über 80-Jährigen zugenommen haben (Abb. 2).

### Verordnungen nach Facharztkontakt

Versicherte mit orthopädischem Facharztkontakt erhielten am häufigsten PT (64 % bei RA vs. 49 % der internistisch-rheumatologisch betreuten Versicherten mit RA). Die Angaben für alle Krankheitsbilder sind in Tab. 4 berichtet.

**Table 4:** 

	RA	axSpA	PsA	SLE
Hausärztlich	46	49	47	43
Rheumatologisch	49	57	49	44
Orthopädisch	64	67	64	62
Andere Fachärzte	49	52	50	44

*RA* rheumatoide Arthritis, *axSpA* axiale Spondyloarthritis, *PsA* Psoriasisarthritis, *SLE* systemischer Lupus erythematodes

### Anzahl der Verordnungen

Die Anzahl der verschriebenen Einheiten hat sich seit 2010 nicht wesentlich verändert. Im Jahr 2010 erhielten von allen Versicherten mit mindestens 1 PT-Verordnung und mit rheumatologischem Kontakt 25 % bis zu 6 Einheiten verordnet, 50 % 7 bis 23 Einheiten und 25 % 24 oder mehr Einheiten, im Median 12 im Jahr, im Mittel 21 im Jahr (Standardabweichung 26). Im Jahr 2020 war für Versicherte mit rheumatologischem Kontakt der Median identisch, 25 % erhielten 30 oder mehr Einheiten, im Mittel wurden 25 (Standardabweichung 29) Einheiten verordnet (s. Tabelle S3).

## Diskussion

Die Auswertung aktueller Krankenkassendaten der BARMER zeigt, dass etwas weniger als die Hälfte aller Versicherten mit Diagnose einer RA, axSpA, PsA oder SLE im Jahr 2020 eine ambulante Verordnung aus dem Bereich der physikalischen Medizin erhalten hat. Im Vergleich zu den Vorjahren bis 2005 hat sich weder der Anteil an Versicherten mit PT-Verordnung noch die Anzahl der Behandlungen wesentlich verändert. Dies unterscheidet sich von Daten aus der gesamtdeutschen Population der gesetzlich Versicherten, in der die Anzahl der Verordnungen im Zeitraum 2004 bis 2014 zugenommen hat [5].

Bereits die PROCLAIR-Studie, in der eine Stichprobe von Versicherten zusätzlich nach ihrem Krankheitsstatus befragt worden war, hatte gezeigt, dass viele Versicherte mit RA keine ambulante PT erhalten [6]. Anscheinend hat sich die bestehende extrabudgetäre Verordnungsmöglichkeit nicht auf die Verordnungszahlen ausgewirkt. Die Möglichkeit, PT auch längerfristig zu verordnen, ist möglicherweise bei einem Teil der betreuenden Mediziner noch unzureichend bekannt [1]. Unter Berücksichtigung einer regresssicheren Verordnung muss diese sorgfältig aus dem Heilmittelkatalog ausgewählt und dokumentiert werden. Hierzu bietet die Rheumaakademie gezielte Weiterbildungswochenenden an.

Die Inanspruchnahme von PT ist insgesamt deutlich höher als in der deutschen Gesamtbevölkerung. Daten der Studie zur Gesundheit Erwachsener in Deutschland (DEGS) von 2008 bis 2011 zeigen, dass 27 % der Frauen und 20 % der Männer Verordnungen der physikalischen Medizin erhalten haben. Auch hier nahm der Anteil mit höherem Alter zu, in allen Altersgruppen nahmen mehr Frauen als Männer PT in Anspruch [7].

Die Behandlungsanzahl kann zukünftig variabler abgestimmt werden

Die Daten zeigen auch, dass Versicherte mit rheumatologischem Kontakt seltener PT verordnet bekamen als Personen, die in orthopädischer Betreuung waren. Dieses wurde bereits in der PROCLAIR-Studie offensichtlich [6]. Eine Zunahme ist lediglich bei manueller Therapie und Ergotherapie zu beobachten, während die klassische Massagetherapie kaum noch eine Rolle spielt. Gerade in den jungen Altersgruppen sind die Verordnungen rückläufig, während die Älteren, v. a. die Hochaltrigen, und über alle Altersgruppen hinweg die Frauen häufiger Maßnahmen der PT verordnet bekommen. Hierbei ist zu berücksichtigen, dass in den Abrechnungsdaten nur die von den Patienten auch eingelösten Rezepte erscheinen. Daher ist es nicht auszuschließen, dass jüngere Personen und Männer diese durchaus verordnet bekommen, aber seltener einlösen. Es ist auch möglich, dass die Jüngeren heute häufiger eigenständige Bewegungsangebote (Fitnesskurse/-studio etc.) wahrnehmen, als klassische PT in Anspruch zu nehmen. Dennoch bleiben die insgesamt niedrigen und über die Jahre weiter rückläufigen (eingelösten) Verordnungen bei jungen Menschen mit rheumatischen Erkrankungen auffällig.

Die Corona-Pandemie im Jahr 2020 hat zu keinem übermäßigen Einbruch der Verordnungen geführt, im Vergleich zu 2019 sind sie aber leicht gesunken. Seit dem 01.01.2021 ist die aktualisierte Richtlinie der Heilmittelverordnung in Kraft getreten [3]. Die Regelungen zum langfristigen Heilmittelbedarf bleiben in dieser erhalten und werden für Menschen mit entzündlich rheumatischen Erkrankungen über die Liste der besonderen Versorgungsbedarfe abgedeckt. Vieles ist übersichtlicher geworden, v. a. die Behandlungsanzahl kann zukünftig variabler abgestimmt werden.

Der Anteil an Versicherten mit einer cs/bDMARD-Therapie ist v. a. bei RA und axSpA sehr niedrig. Dies ist aus Studien zur medikamentösen Therapie bekannt [8–10] und lässt sich auf die niedrige Verordnungsrate bei fehlendem fachärztlichem Kontakt, den hohen Anteil an älteren Versicherten und den hohen Anteil an seronegativen Diagnosen zurückführen.

### Limitationen

Unterschiede in den Versichertenstrukturen der gesetzlichen Krankenversicherungen limitieren die Repräsentativität der Versicherungsdaten [11]. Die BARMER hat im Vergleich zu anderen Krankenkassen einen überdurchschnittlich hohen Anteil an älteren Frauen, und auch die Häufigkeit von Begleiterkrankungen unterscheidet sich zu anderen Kassendaten [12]. Durch die Auswertung nach Alters- und Geschlechtergruppen haben wir dies berücksichtigt, aber der Gesamtanteil an Verordnungen sollte nicht als repräsentativ für die deutsche Bevölkerung betrachtet werden. Die auf die deutsche Population der gesetzlich Versicherten nach Alter und Geschlecht standardisierten Daten unterscheiden sich jedoch kaum von den Rohdaten – Unterschiede zur deutschen Gesamtbevölkerung könnten also höchstens durch andere Merkmale als Alter und Geschlecht begründet sein.

Aus den Kassendaten geht nicht hervor, für welche Indikation die PT verordnet wurde. Es ist also naheliegend, dass auch andere nichtentzündliche Begleiterkrankungen am Bewegungsapparat für die PT-Verordnungen ausschlaggebend waren, daher wurden die wichtigsten Begleitdiagnosen, die häufig mit PT-Verordnungen einhergehen, berichtet.

In der Kerndokumentation der Rheumazentren werden die Rheumatologen gefragt, ob bei ihren Patienten in den letzten 12 Monaten Krankengymnastik erfolgt ist [13]. Diese Angaben sind (außer bei axSpA) niedriger als in den Kassendaten: Im Jahr 2019 wurde bei 31 % (RA), 48 % (axSpA), 30 % (PsA) und 18 % (SLE) Krankengymnastik dokumentiert (Angaben aus der jährlichen Standardpräsentation der Kerndokumentation). Vor allem bei SLE scheinen andere Indikationen oder unabhängig von der rheumatologischen Versorgung erfolgte PT häufiger zu sein. In der Kerndokumentation wird auch abgefragt, ob die Patienten in den letzten 12 Monaten am Funktionstraining teilgenommen haben. Dies traf 2019 für 2 % (SLE) bis 5 % (axSpA) der Patienten zu (Angaben aus der jährlichen Standardpräsentation).

Vergleicht man die Verordnungshäufigkeit bei entzündlich rheumatischen Erkrankungen mit Daten von Versicherten mit nichtentzündlichen Gelenkerkrankungen, zeigen sich hier ähnliche Ergebnisse: Nur knapp jeder zweite Versicherte mit einer Arthrose erhielt im Jahr vor einem Knie- oder Hüftgelenkersatz eine PT-Behandlung [14]. Auch bei Arthrose blieb ca. ein Drittel der Patienten mit starken Funktionseinschränkungen bzw. Schmerzen ohne PT [15]. Es sollte aber auch berücksichtigt werden, dass einige Patienten PT-Maßnahmen aus den verschiedensten Gründen nicht wahrnehmen möchten, obwohl sie ihnen ärztlicherseits empfohlen werden.

## Fazit für die Praxis

- Die Verordnung von physikalischer Therapie (PT) bei Menschen mit entzündlich rheumatischen Erkrankungen ist seit vielen Jahren auf gleichbleibendem Niveau.
- Die Möglichkeiten einer Langzeitverordnung bei entzündlich rheumatischen Erkrankungen haben offenbar zu keinem relevanten Anstieg der Verordnungen geführt.
- Auch vor dem Hintergrund der großen Erfolge der medikamentösen Therapie bleiben bei Personen mit entzündlich rheumatischen Erkrankungen – auch aufgrund von Multimorbidität – viele Einschränkungen, die der physikalischen Medizin zugänglich sind. Die entsprechenden Möglichkeiten können noch stärker ausgeschöpft werden.

## Supplementary Information




