# Supplementary material for: Prescription frequency of physical therapy for inflammatory rheumatic diseases
Source: Z Rheumatol. 2022 Mar 22;81(5):360–8. [Article in German] doi: 10.1007/s00393-022-01180-x (PMC9156497; doi:10.1007/s00393-022-01180-x)
Supplement: Supplementary file 1 [file 393_2022_1180_MOESM1_ESM.pdf]

Zusatzmaterial

**Tabelle S1** ATC Codes der Medikamentengruppen		
Medikamentengruppe	Jahr	ATC-Codes
Glukokortikoide	2006, 2020	H02AB01, H02AB02, H02AB03, H02AB04, H02AB05, H02AB06, H02AB07, H02ABX
NSAR	2006, 2020	M01A
csDMARDs	2006, 2020	A07EC01, C02KX01, M01C, P01BA01, P01BA02, M01CX02, M01CX01, L04AX03, L04AA13, L04AX01, L01AA01, L04AA01, L04AA06, L04AA05
bDMARDs	2006	L04AA11, L04AA12, L04AA17, L04AA14
bDMARDs	2020	L04AB, L04AA24, L04AC03, L04AC07, L01CX02, L04AA26, L04AC10, L04AC13, L04AC15, L04AC08, L04AC05
tsDMARDs	2020	L04AA32, L04AA29, L04AA37

Tabelle S2 Anteil der Personen (%) mit mindestens einer physiotherapeutischen Verordnung im jeweiligen Jahr, nach Krankheitsbildern

	N	Physikalische Therapie (alle)	Kranken-gymnastik	Massagen	Manuelle Therapie	Thermo-therapie	Elektro-therapie	Ergo-therapie
RA								
2005	78834	46	35	13	7	17	3.4	4
2006	88679	45	35	12	8	15	3.0	6
2007	91468	46	35	11	8	15	2.8	7
2008	95496	46	35	10	9	15	2.9	7
2009	99125	45	35	9	10	14	2.7	8
2010	104312	46	35	9	11	14	2.6	8
2011	106293	47	36	8	11	14	2.6	9
2012	110175	47	36	7	12	14	2.4	9
2013	115140	47	36	7	13	13	2.4	10
2014	119262	48	37	6	14	13	2.3	10
2015	122412	48	37	6	15	13	2.2	10
2016	141468	43	33	4	14	11	2.0	9
2017	143793	48	38	4	15	11	2.4	10
2018	145128	48	39	4	15	11	2.4	11
2019	146404	49	39	3	15	11	2.3	11
2020	144921	46	37	3	14	10	2.0	11
		Physikalische Therapie (alle)	Kranken-gymnastik	Massagen	Manuelle Therapie	Thermo-therapie	Elektro-therapie	Ergo-therapie
axSpA								
2005	15448	50	38	16	8	19	3	3
2006	17521	48	37	14	9	17	3	3
2007	17961	48	37	13	9	17	2	4
2008	18754	48	36	12	10	16	2	5
2009	19321	47	36	11	11	16	2	5
2010	20418	48	36	10	12	16	2	6
2011	20860	49	38	9	13	15	2	6
2012	21851	49	37	8	14	15	2	6
2013	22948	49	37	8	15	15	2	7
2014	23830	51	39	7	16	14	2	7
2015	24238	50	38	6	16	14	2	7

2016	28064	45	35	5	15	12	2	6
2017	28606	50	39	4	16	13	2	7
2018	29040	51	40	4	16	13	2	7
2019	29499	51	40	3	17	12	2	8
2020	29249	49	38	3	16	11	2	8
		Physikalische Therapie (alle)	Kranken-gymnastik	Massagen	Manuelle Therapie	Thermo-therapie	Elektro-therapie	Ergo-therapie
PsA								
2005	2998	43	33	12	8	16	2,9	3,6
2006	3945	44	34	11	8	15	3,3	4,1
2007	4587	45	34	11	10	15	3,1	4,9
2008	5416	44	34	10	9	15	2,8	5,7
2009	6113	45	34	10	11	15	2,5	6,6
2010	6990	45	34	9	12	15	2,8	7,5
2011	8626	46	34	8	13	15	2,8	7,6
2012	9923	45	33	7	14	14	2,8	7,9
2013	11110	46	34	6	15	13	2,3	8,9
2014	12128	47	35	6	16	14	2,2	8,4
2015	12857	48	35	6	16	14	2,2	8,5
2016	15679	43	32	4	15	12	2	8,6
2017	16971	49	37	4	17	13	2,6	9
2018	17930	49	38	4	17	13	2,3	10,1
2019	19090	49	38	3	17	13	2,2	10,4
2020	19760	46	36	2	16	11	2	10,6
		Physikalische Therapie (alle)	Kranken-gymnastik	Massagen	Manuelle Therapie	Thermo-therapie	Elektro-therapie	Ergo-therapie
SLE								
2005	2569	42	29	14	8	16	4	4
2006	2863	42	30	12	8	14	3	5
2007	2951	42	31	12	9	15	3	6
2008	3209	42	31	10	10	13	3	7
2009	3345	42	30	9	11	14	3	7
2010	3528	44	32	9	12	14	3	8
2011	3620	44	32	9	13	15	3	9
2012	3855	43	31	7	13	13	2	9
2013	4109	43	32	7	13	13	3	9
2014	4247	45	33	6	14	13	2	10
2015	4444	44	33	5	14	11	2	10
2016	5055	40	29	4	14	10	2	8
2017	5329	45	34	4	15	11	3	10
2018	5490	45	34	3	15	11	3	10
2019	5483	45	34	3	15	10	2	11
2020	5458	43	33	3	14	9	2	9
RA rheumatoide Arthritis, axSpA axiale Spondyloarthritis, PsA Psoriasisarthritis, SLE systemischer Lupus erythematodes								

**Tabelle S3** Anzahl der verschriebenen physikalische Therapie Termine pro Patient:in				
Fachrichtung	Jahr	Mittelwert (Standardabw.)	Median	Quartil 1; Quartil 3
Alle	2010	25 (33)	14	6; 30
Allgemeinmedizin	2010	21 (30)	12	6; 24
Rheumatologie	2010	21 (26)	12	6; 24
Orthopädie	2010	17 (19)	12	6; 20
Andere Fachrichtung	2010	14 (25)	6	2; 14
Alle	2020	25 (35)	14	6; 32
Allgemeinmedizin	2020	20 (33)	12	6; 24
Rheumatologie	2020	25 (29)	12	6; 30
Orthopädie	2020	17 (20)	12	6; 20
Andere Fachrichtung	2020	16 (28)	6	10; 18

Abbildung S1 Verordnungen von Physikalischer Therapie in den Jahren 2005 – 2020, standardisiert nach Alter und Geschlecht auf die Population der gesetzlich Krankenversicherten in Deutschland im Jahr 2020.

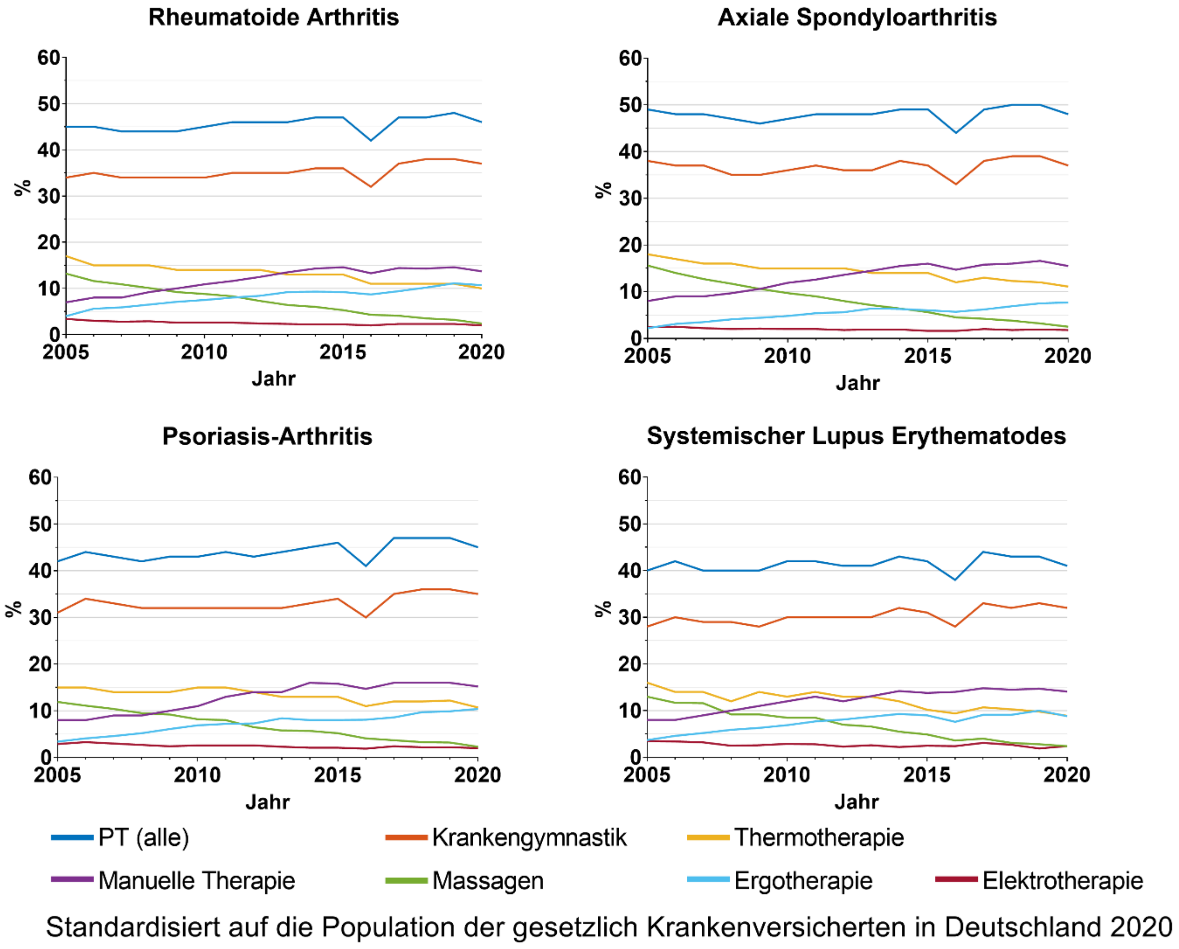

Abbildung S2 Verordnungen von Physikalischer Therapie in den Jahren 2005 – 2020, standardisiert nach Alter und Geschlecht auf die jeweilige Population Barmer-Versicherter mit der entsprechenden Diagnose in 2020.

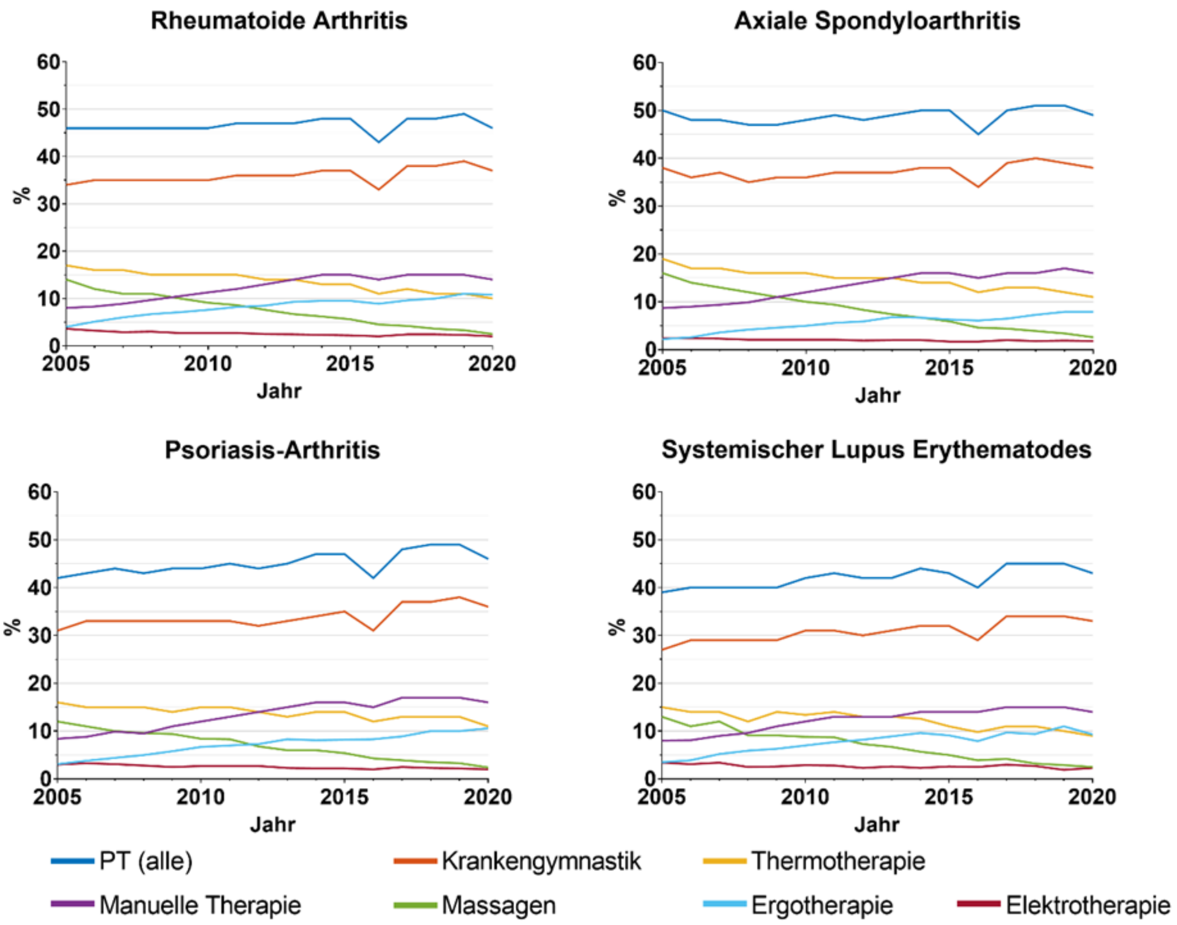

Standardisiert auf die jeweilige Patientenpopulation von 2020
